# The prognostic significance of celiac lymph node metastasis in patients with locally advanced esophageal squamous cell carcinoma receiving curative concurrent chemoradiotherapy

**DOI:** 10.18632/oncotarget.21878

**Published:** 2017-10-16

**Authors:** Yen-Hao Chen, Hung-I Lu, Yu-Ming Wang, Chien-Ming Lo, Shang-Yu Chou, Cheng-Hua Huang, Li-Hsueh Shih, Su-Wei Chen, Shau-Hsuan Li

**Affiliations:** ^1^ Department of Hematology-Oncology, Kaohsiung Chang Gung Memorial Hospital and Chang Gung University College of Medicine, Kaohsiung, Taiwan; ^2^ Graduate Institute of Clinical Medical Sciences, College of Medicine, Chang Gung University, Taoyuan, Taiwan; ^3^ School of Medicine, Chung Shan Medical University, Taichung, Taiwan; ^4^ Department of Thoracic & Cardiovascular Surgery, Kaohsiung Chang Gung Memorial Hospital and Chang Gung University College of Medicine, Kaohsiung, Taiwan; ^5^ Department of Radiation Oncology, Kaohsiung Chang Gung Memorial Hospital and Chang Gung University College of Medicine, Kaohsiung, Taiwan; ^6^ Department of Nursing, Kaohsiung Chang Gung Memorial Hospital, Kaohsiung, Taiwan; ^7^ Department of Anesthesia, Kaohsiung Medical University Hospital, Kaohsiung, Taiwan

**Keywords:** celiac lymph node, esophageal cancer, squamous cell carcinoma, concurrent chemoradiotherapy

## Abstract

**Background:**

To evaluate the clinical outcomes of celiac lymph node (LN) metastasis in patients with locally advanced esophageal squamous cell carcinoma (ESCC) receiving curative concurrent chemoradiotherapy (CCRT).

**Materials and Methods:**

A total of 375 stage III ESCC patients were identified, including 51 patients with celiac LN metastasis and 324 patients without celiac LN metastasis. Among these 324 patients without celiac LN metastasis, 51 were matched with the 51 patients with celiac LN metastasis using the propensity score matching method.

**Results:**

Overall, the celiac LN metastasis group had worse progression-free survival (PFS) and overall survival (OS) than the non-celiac LN metastasis group and the matched non-celiac LN metastasis group. For the ESCC patients with celiac LN metastasis, lower third ESCC was significantly associated with superior PFS and OS. For patients with upper/middle third ESCC, the celiac LN metastasis group had worse PFS and OS than the non-celiac LN metastasis group and the matched non-celiac LN metastasis group. For patients with lower third ESCC, there were no significant differences in PFS and OS between these groups.

**Conclusions:**

Our study suggests celiac LN metastasis is a poor prognostic factor for locally advanced ESCC patients receiving curative CCRT. Among these ESCC patients with celiac LN metastasis, tumor location is a strongly prognostic factor, indicating patients with lower third ESCC have better PFS and OS than those with upper/middle third ESCC. The 6^th^ American Joint Committee on Cancer staging system seems more favorable than 7^th^ edition in the definition of celiac LNs for those patients.

## INTRODUCTION

Esophageal squamous cell carcinoma (ESCC) is an aggressive disease with an increasing incidence worldwide, and is the ninth leading cause of cancer deaths in Taiwan [1]. The risk factors of ESCC include long-term use of tobacco and alcohol, betel quid chewing, chronic mucosal irritation, hot beverages and food consumption, achalasia, esophageal web, and upper aerodigestive cancer history [2–4]. Most of patients with ESCC are in lower socioeconomic status and some patients have family history of esophageal cancer [5–7]. The majority of ESCC patients have locally advanced disease when they are diagnosed, and more than half of patients with locally advanced disease are clinically unresectable. For those patients who are unresectable, concurrent chemoradiotherapy (CCRT) is one of the standard therapies. Nonetheless, in spite of significant improvements having been made in chemotherapy and radiotherapy, the outcomes of such ESCC patients remain poor [8–12].

Lymphatic metastasis in cases of esophageal cancer can spread bidirectionally and reach remote locations ranging from the cervical to celiac lymph nodes (LNs), with celiac LN metastasis occurring frequently in locally advanced ESCC patients. In the 6^th^ edition of the American Joint Committee on Cancer (AJCC) staging system, celiac LNs are defined as non-regional LNs in cases of thoracic esophageal cancer, in addition to being classified as M1a stage LNs in cases of lower third esophageal cancer and as M1b stage LNs in cases of upper and middle third esophageal cancer [13]. However, the 7^th^ AJCC staging system re-defines celiac LNs as regional LNs and removes the M1a and M1b classifications [14]. Furthermore, N stages are subclassified based on the absolute number of positive LNs instead of the presence of regional LN involvement. At the same time, several studies have shown that celiac LN metastasis does not compromise the clinical outcomes of patients who have undergone esophagectomy [15–17]. Moreover, Tachimori *et al*. reported that the factor associated with LN metastasis that was most predictive of postoperative survival was not the area of the involved nodes, but the number of involved LNs [17]. However, ESCC patients with celiac LN metastasis have not been enrolled in most phase III clinical trials; hence, for unresectable locally advanced esophageal cancer patients, the prognostic significance of celiac LN metastasis is still unclear [18, 19]. In the present study, we retrospectively analyzed the records of locally advanced ESCC patients, including those with celiac LN metastasis, who underwent CCRT as curative treatment in our hospital, with the aim of our study being to evaluate the prognostic significance of celiac LN metastasis in locally advanced ESCC patients receiving curative CCRT.

## RESULTS

### Patient characteristics

We retrospectively reviewed our ESCC database, and 375 locally advanced stage III ESCC patients who received curative CCRT were identified, including 51 ESCC with celiac LN metastasis. Of these 51 ESCC patients, 48 were men and 3 were women, and they had a mean age of 56 years (range: 42 to 80 years). The 1-year and 2-year survival rates of these patients were 50% and 21%, respectively. The tumor T status was revealed to be T2 in two (4%) patients, T3 in 23 (45%) patients, and T4 in 26 (51%) patients, while the node N status was found to be N1 in two (4%) patients, N2 in 17 (33%) patients, and N3 in 32 (63%) patients. Additional analyses conducted according to AJCC 7^th^ staging system indicated stage IIIA tumors for one (2%) patient, stage IIIB tumors for 10 (20%) patients, and stage IIIC tumors for 40 (78%) patients. Further analyses of histological grades showed a grade 1 lesion in 5 (10%) patients, grade 2 lesion in 23 (45%) patients, and grade 3 lesion in 23 (45%) patients. The primary tumor location was found to be the upper esophagus in 6 (12%) patients, the middle esophagus in 18 (35%) patients, and the lower esophagus in 27 (53%) patients. The clinicopathological parameters of these patients are shown in Table 1.

**Table 1:** Characteristics of 51 esophageal squamous cell carcinoma patients with celiac lymph node metastasis

Characteristics	
Age	56 years old (42-80)
Sex	
Male	48 (94%)
Female	3 (6%)
T status	
1	0 (0%)
2	2 (4%)
3	23 (45%)
4	26 (51%)
N status	
0	0 (0%)
1	2 (4%)
2	17 (33%)
3	32 (63%)
Tumor stage	
IIIA	1 (2%)
IIIB	10 (20%)
IIIC	40 (78%)
Grade	
1	5 (10%)
2	23 (45%)
3	23 (45%)
Location	
Upper	6 (12%)
Middle	18 (35%)
Lower	27 (53%)

### Comparison between ESCC with and without celiac LN metastasis

These 375 stage III locally advanced ESCC patients were divided into two groups: a celiac LN metastasis group (N=51) and a non-celiac LN metastasis group (N=324). The baseline characteristics did not differ significantly among these two groups, apart from tumor stage (P=0.002) and tumor location (P<0.001). More specifically, the celiac LN metastasis group had a greater proportion of cases with stage IIIC disease and lower third tumor location compared to the non-celiac LN metastasis group.

Among the 324 ESCC patients without celiac LN metastasis, 51 matched patients were identified for comparison with the celiac LN metastasis group using the propensity score matching method. Age, tumor stage, grade, and location were all matched so that there was no statistical difference between these two groups except for with respect to the sex ratios of the two groups. The clinicopathological parameters of these patients are shown in Table 2.

**Table 2:** Clinicopathological parameters in 375 stage III locally advanced esophageal squamous cell carcinoma patients with/without celiac lymph node metastasis receiving CCRT

Characteristics	Celiac LN group (N=51)	Non-celiac LN group (N=324)	P value
Age			
< 60 years	36 (71%)	234 (72%)	0.87
≥ 60 years	32 (29%)	90 (28%)	
Sex			
Male	48 (94%)	316 (98%)	0.20
Female	3 (6%)	8 (2%)	
Tumor stage			
IIIA	1 (2%)	86 (27%)	0.002^*^
IIIB	10 (20%)	40 (12%)	
IIIC	40 (78%)	198 (61%)	
Grade			
1	5 (10%)	50 (15%)	0.14
2	23 (45%)	102 (31%)	
3	23 (45%)	172 (54%)	
Location			
Upper	6 (12%)	102 (31%)	<0.001^*^
Middle	18 (35%)	139(43%)	
Lower	27 (53%)	83 (26%)	
**Characteristics**	**Celiac LN group (N=51)**	**#Matched non-celiac LN group (N=51)**	**P value**
Age			
< 60 years	36 (71%)	36 (71%)	1.0
≥ 60 years	32 (29%)	32 (29%)	
Sex			
Male	48 (94%)	51 (100%)	0.08
Female	3 (6%)	0 (0%)	
Tumor stage			
IIIA	1 (2%)	1 (2%)	1.0
IIIB	10 (20%)	10 (20%)	
IIIC	40 (78%)	40 (78%)	
Grade			
1	5 (10%)	5 (10%)	1.0
2	23 (45%)	23 (45%)	
3	23 (45%)	23 (45%)	
Location			
Upper	6 (12%)	6 (12%)	1.0
Middle	18 (35%)	18 (35%)	
Lower	27 (53%)	27 (53%)	

LN: lymph node; CCRT: concurrent chemoradiotherapy; #Using propensity score matching method. ^*^Statistically significant.

**Figure 1:**
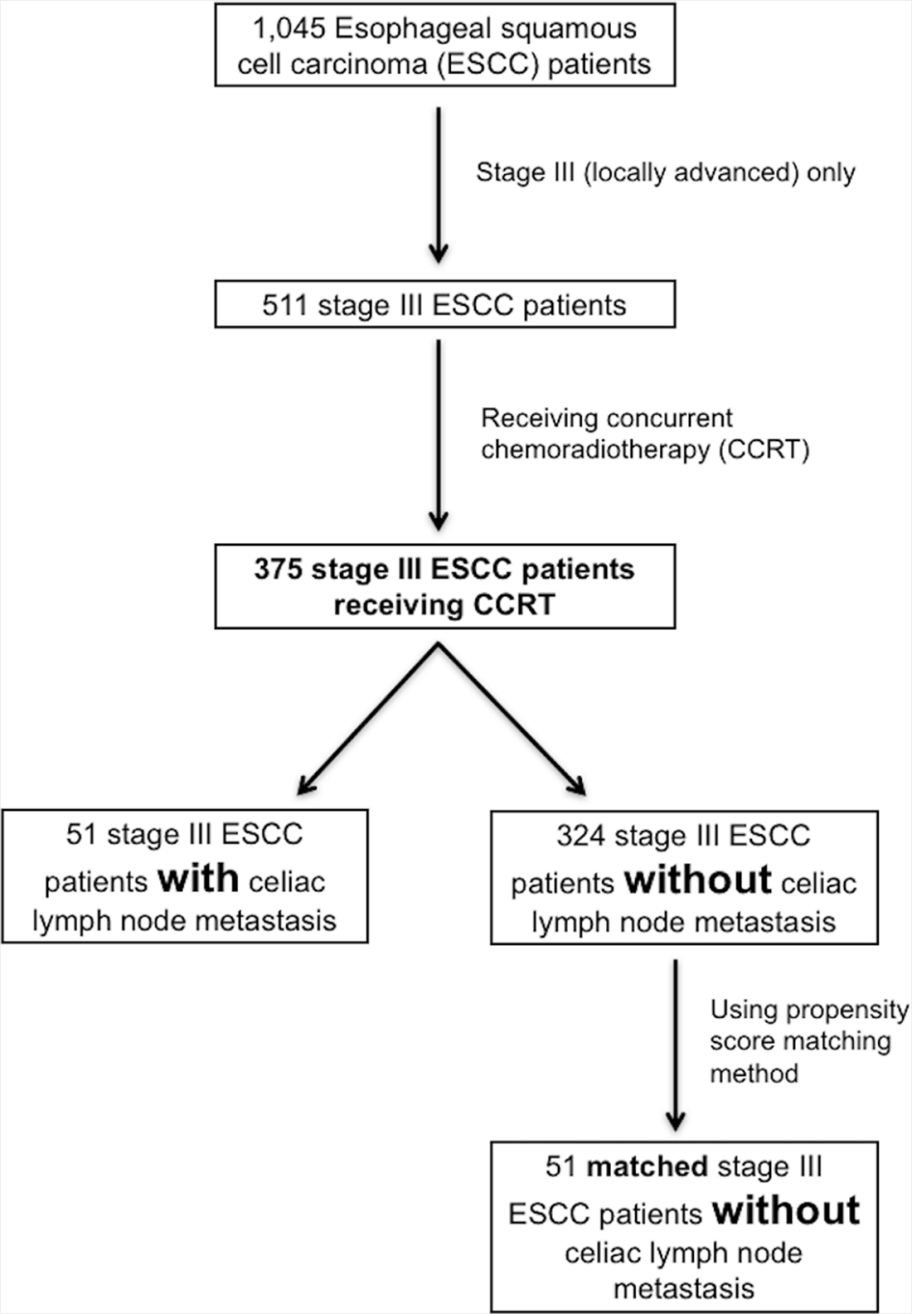
The progression-free survival (PFS) and overall survival (OS) curves of esophageal squamous cell carcinoma patients with/without celiac LN metastasis **(A)** PFS, celiac LN metastasis group versus non-celiac metastasis LN group **(B)** PFS, celiac LN metastasis group versus matched non-celiac LN metastasis group using the propensity score matching method. **(C)** OS, celiac LN metastasis group versus non-celiac LN metastasis group **(D)** OS, celiac LN metastasis group versus matched non-celiac metastasis LN group using the propensity score matching method. LN: lymph node.

The celiac LN metastasis group (N=51) was found to have worse progression-free survival (PFS) than the non-celiac LN metastasis group (N=324, 6.4 months versus 10.1 months, P=0.004, Figure 1A) and the matched non-celiac LN metastasis group (N=51, 6.4 months versus 10.0 months, P=0.037, Figure 1B). In addition, the celiac LN metastasis group (N=51) was also found to have worse overall survival (OS) compared to the non-celiac LN metastasis group (N=324, 13.5 months versus 18.3 months, P=0.037, Figure 1C) and the matched non-celiac LN metastasis group (N=51, 13.5 months versus 17.2 months, P=0.039, Figure 1D).

Among these 375 stage III locally advanced ESCC patients, 361 patients were mentioned to have treatment failure, including 45 patients in the celiac LN metastasis group and 316 patients in the non-celiac LN metastasis group. The celiac LN metastasis group was found to have higher percentage of distant metastasis than the non-celiac LN metastasis group (53% versus 27%, P<0.001).

### Clinical outcomes of ESCC patients with celiac LN metastasis receiving curative CCRT

In the analysis of PFS, there were no significant differences in terms of age, sex, and tumor grade in a univariate analysis. Meanwhile, the total of 25 patients with T1-3 status had significantly superior PFS compared to the 26 patients with T4 status (8.9 months versus 3.8 months, P=0.036), and the 19 patients who had N0-2 status had better PFS than the other 32 patients with N3 status (11.2 months versus 4.2 months, P=0.009). The 11 stage IIIA and IIIB patients had superior PFS in comparison with the 40 stage IIIC patients (12.6 months versus 4.3 months, P=0.027), and the total of 27 patients who had a tumor in the lower third of the esophagus had better PFS than the other 24 patients with a tumor located in the upper or middle third of the esophagus (8.9 months versus 4.3 months, P=0.045). Multivariate analysis revealed N0-2 status (P=0.013, hazard ratio: 0.44, 95% confidence interval: 0.23-0.84) represented the independent predictive factors of superior PFS.

In the analysis of OS, there were no significant differences in overall survival in terms of age, sex, T status, N status, and tumor stage in a univariate analysis. Meanwhile, the total of 29 ESCC grade 1 and 2 patients had significantly superior OS compared to the 22 ESCC grade 3 patients (19.1 months versus 10.0 months, P=0.030), and the 27 patients who had a tumor in the lower third of the esophagus had better OS than the other 24 patients with a tumor located in the upper or middle third of the esophagus (15.1 months versus 9.2 months, P=0.023). According to a multivariate comparison, a tumor grade of 1/2 (P=0.004, hazard ratio: 0.34, 95% confidence interval: 0.16-0.71) and tumor location in the lower third of the esophagus (P=0.015, hazard ratio: 0.41, 95% confidence interval: 0.20-0.84) represented the independent predictive factors of superior OS. The univariate and multivariate analyses of PFS and OS in 51 ESCC patients with celiac LN metastasis are shown in Table 3.

**Table 3:** Univariate and multivariate analysis of progression-free survival and overall survival in in 51 esophageal squamous cell carcinoma patients with celiac lymph node metastasis

Characteristics	No. of patients	Univariate analysis		Multivariate analysis		Univariate analysis		Multivariate analysis	
		Median PFS (months)	P value	HR (95% CI)	P value	Median OS (months)	P value	HR (95% CI)	P value
Age									
< 60 years	36 (71%)	6.1	0.99			12.0	0.68		
≥ 60 years	15 (29%)	7.3				13.6			
Sex									
Male	48 (94%)	6.1	0.07			13.5	0.27		
Female	3 (6%)	7.3				9.2			
T status									
1 + 2 + 3	25 (49%)	8.9	0.036^*^			15.1	0.16		
4	26 (51%)	3.8				10.5			
N status									
0 + 1 + 2	19 (37%)	11.2	0.009^*^	0.44 (0.23-0.84)	0.013^*^	13.6	0.12		
3	32 (63%)	4.2				10.5			
Tumor stage									
IIIA + IIIB	11 (22%)	12.6	0.027^*^			20.3	0.41		
IIIC	40 (78%)	4.3				12.0			
Grade									
1 + 2	29 (57%)	6.1	0.19			19.1	0.030^*^	0.34 (0.16-0.71)	0.004^*^
3	22 (43%)	6.4				10.0			
Location									
Upper + Middle	24 (47%)	4.3	0.045^*^			9.2	0.023^*^		
Lower	27 (53%)	8.9				15.1		0.41 (0.20-0.84)	0.015^*^

PFS: progression-free survival; OS: overall survival; HR: hazard ratio; CI: confidence interval ^*^Statistically significant.

### Clinical impact of celiac LN metastasis for the different tumor locations in the esophagus

**Figure 2:**
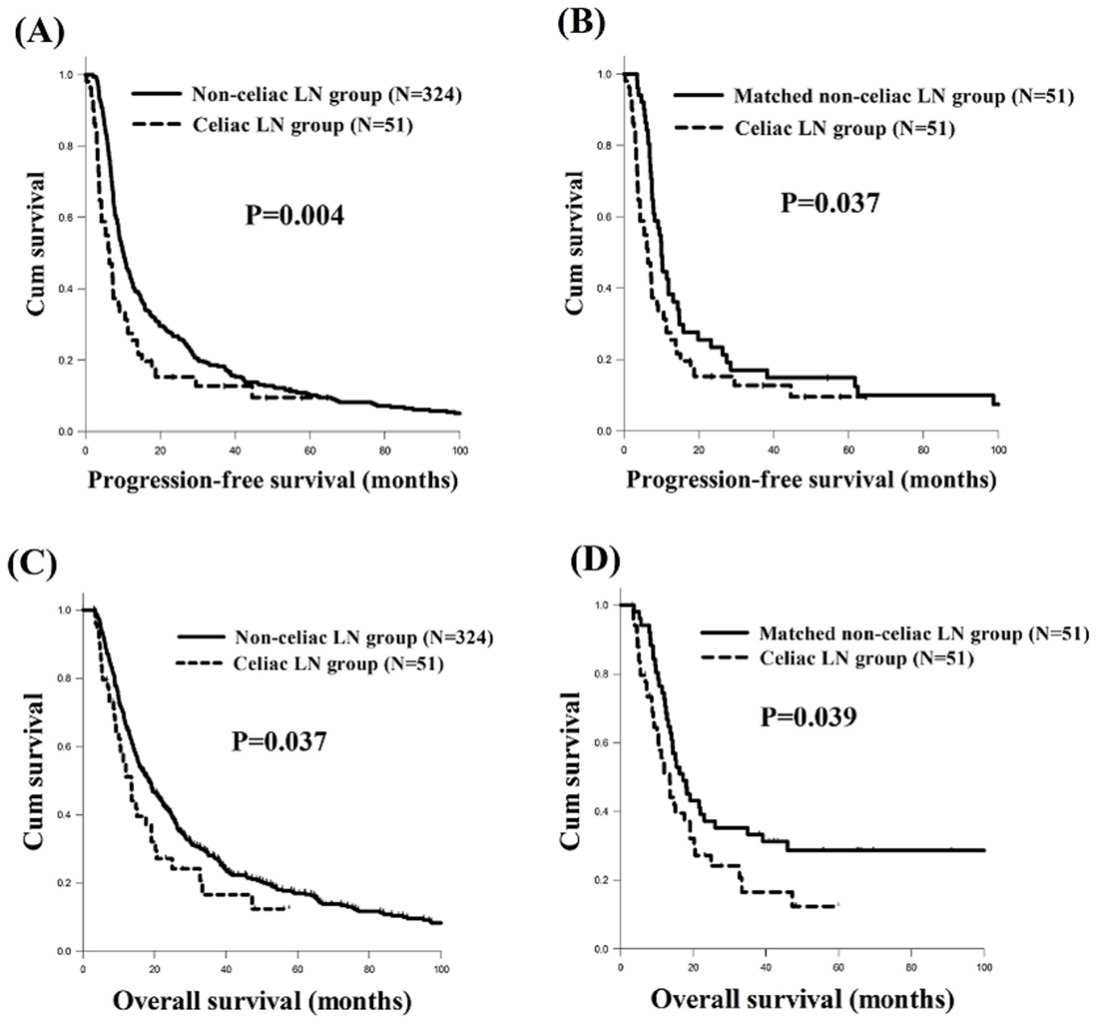
Comparison of survival curves of esophageal squamous cell carcinoma patients harboring celiac lymph node metastasis with different tumor locations **(A)** progression-free survival, upper/middle thirds versus lower third. **(B)** overall survival, upper/middle thirds versus lower third.

**Figure 3:**
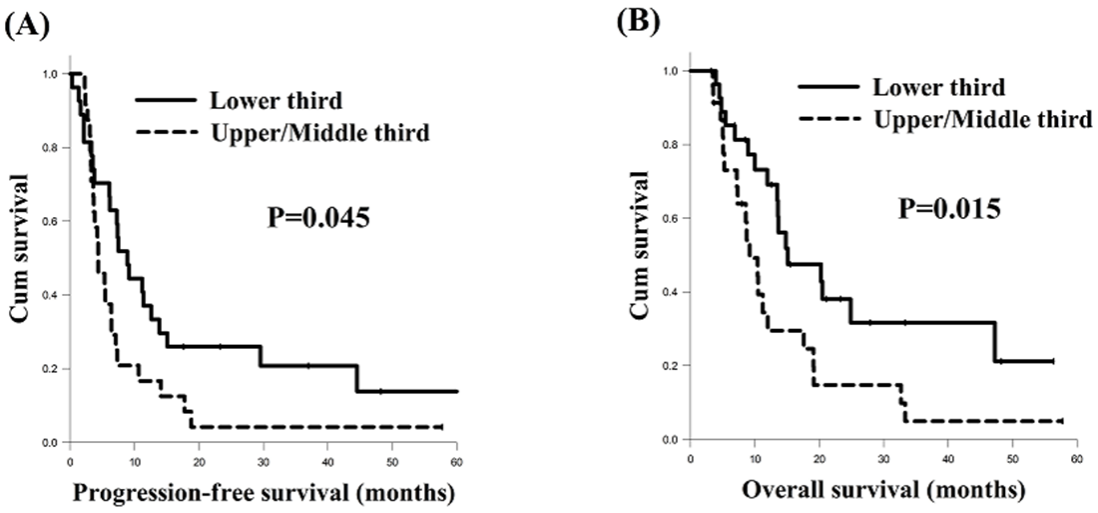
Comparison of progression-free survival curves of esophageal squamous cell carcinoma patients with different tumor locations **(A)** Upper/middle thirds, celiac LN metastasis group versus non-celiac LN metastasis group **(B)** Lower third, celiac LN metastasis group versus non-celiac LN metastasis group **(C)** Upper/middle thirds, celiac LN metastasis group versus matched non-celiac LN metastasis group **(D)** Lower third, celiac LN metastasis group versus matched non-celiac LN metastasis group. LN: lymph node.

**Figure 4:**
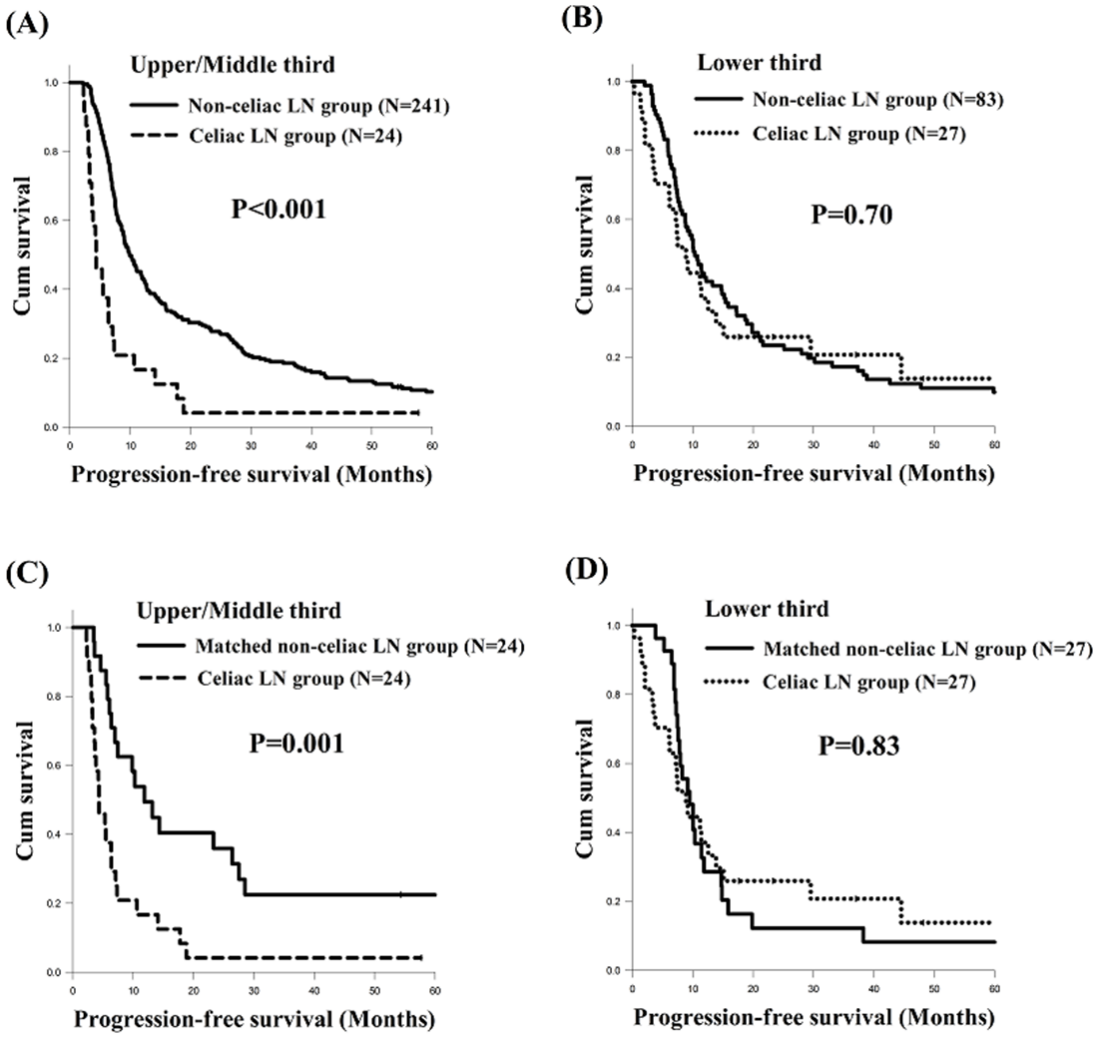
Comparison of overall survival curves of esophageal squamous cell carcinoma patients with different tumor locations **(A)** Upper/middle thirds, celiac LN metastasis group versus non-celiac LN metastasis group **(B)** Lower third, celiac LN metastasis group versus non-celiac LN metastasis group **(C)** Upper/middle thirds, celiac LN metastasis group versus matched non-celiac LN metastasis group **(D)** Lower third, celiac LN metastasis group versus matched non-celiac LN metastasis group. LN: lymph node.

In the present study, we found that the tumor location was significantly associated with PFS and OS in ESCC with celiac LN metastasis (Figure 2). Therefore, we also evaluated if there was any significant difference between the celiac LN metastasis group and the non-celiac LN metastasis group in terms of different tumor locations. First, we compared the PFS and OS of the celiac LN metastasis group and the non-celiac LN metastasis group patients with different tumor locations in the esophagus. There were 265 ESCC patients in total who had a tumor located in the upper or middle third of esophagus, including 24 patients in the celiac LN metastasis group and 241 patients in the non-celiac LN metastasis group. Of those patients, those in the celiac LN metastasis group had worse PFS (4.3 months versus 9.9 months, P<0.001, Figure 3A) and OS (9.2 months versus 18.4 months, P=0.001, Figure 4A) than those in the non-celiac LN metastasis group. On the other hand, the other 110 patients, who consisted of 27 patients in the celiac LN metastasis group and 83 patients in the non-celiac LN metastasis group, had lower third ESCC, and there were no significant differences in PFS and OS between those two groups (Figure 3B and Figure 4B). Second, we also compared the PFS and OS of the celiac LN metastasis group and the matched non-celiac LN metastasis group patients with different tumor locations in the esophagus. The survival outcome results were essentially the same as those for the comparison between the celiac LN metastasis group and the overall non-celiac LN metastasis group noted above. That is, among the upper or middle third ESCC patients, the celiac LN metastasis group had worse PFS (4.3 months versus 11.9 months, P=0.001, Figure 3C) and OS (9.2 months versus 23.0 months, P=0.001, Figure 4C) than the matched non-celiac LN metastasis group. For lower third ESCC patients, however, there were no significant differences in PFS and OS between these two groups (Figure 3D and Figure 4D).

## DISCUSSION

Patients with celiac LN metastasis constitute a small portion of the overall population of patients diagnosed with ESCC. To the best of our knowledge, there have been only a few studies that have evaluated and discussed the efficacy and outcomes of the various treatment options for patients with celiac LN metastasis. In the 6^th^ AJCC staging system, celiac LNs are defined as non-regional LNs in cases of thoracic esophageal cancer and are classified as M1a stage LNs in cases of lower third esophageal cancer and as M1b stage LNs in cases of for upper and middle third esophageal cancer [13]. However, in the 7^th^ AJCC staging system, celiac LNs are re-defined as regional LNs, and N stages are subclassified based on the number of positive LNs. Most previous trials did not include the esophageal cancer patients with celiac LN metastasis (M1a or M1b disease) as defined by the 6^th^ AJCC staging system, so the prognostic significance of celiac LN metastasis has not been addressed properly [18–20].

Recently, several studies found that celiac LN metastasis does not compromise clinical outcomes [15, 17]. A Korean study, reported by Cho *et al*., showed metastasis to celiac LN was a significant factor neither for PFS nor OS in ESCC patients receiving neoadjuvant chemoradiotherapy and surgery [15]. Another Japanese study, reported by Tachimori *et al*., revealed the most predictive factor associated with lymph node metastasis for postoperative survival was not the area of involved nodes, but the number of involved nodes in ESCC patients receiving esophagectomy and three-field LN dissection [17]. However, the current study found that celiac LN metastasis is a predictive factor of poor prognosis in ESCC patients receiving curative CCRT. This discrepancy between our study and other previous studies may be related to several factors. First, our study only included cases of locally advanced stage III disease, meaning that surgical resection was not always feasible for these patients. In contrast, the esophageal cancer patients enrolled in previous studies received either neoadjuvant chemoradiotherapy followed by surgery or surgery alone, rather than curative CCRT, indicating relatively early stage in these patients, and making those studies very different from our study. All the ESCC patients in our study received CCRT as a curative treatment, after which surgical resection was indicated in some situations, such as tumor downstaging or a progressive disease that was still resectable. However, the risk of local recurrence or distant metastasis increased if complete remission did not achieve after CCRT, contributing to poor prognosis, but surgery may overcome the disadvantage of CCRT. In Cho's study, most patients received surgery after neoadjuvant chemoradiotherapy, and esophagectomy and three-field LN dissection was performed in all ESCC patients in Tachimori's study. Therefore, the disease statuses and treatment modality of the patients in our study were generally different from those of patients in other studies.

Second, although our study found celiac LN metastasis to be predictive of poor prognosis, the role of celiac LN metastasis differed in the different tumor locations in the esophagus. For upper or middle third ESCC patients, celiac LN metastasis was found to be a strong prognostic factor of poor prognosis compared to patients without celiac LN metastasis, whether in the matched or non-matched group. In contrast, there were no significant differences in overall survival between the lower third ESCC patients with celiac LN metastasis and those lower third ESCC patients without celiac LN metastasis. Given the above findings, it is possible that celiac LN metastasis should be considered as regional LN metastasis for lower third ESCC patients, while being regarded as non-regional LN metastasis for ESCC patients with tumors located in the upper or middle third of the esophagus. However, ESCC patients with celiac LN metastasis were not enrolled in most phase III clinical trials; as such, for patients with unresectable locally advanced esophageal cancer, the prognostic significance of celiac LN metastasis is still unclear [18, 19]. Induction chemotherapy or more powerful therapeutic regimens before CCRT may be a treatment option, and further larger prospective studies would be warranted to establish the optimal treatment for ESCC patients with celiac LN metastasis.

In the 7^th^ AJCC staging system, non-anatomic cancer characteristics, such as tumor location, histologic grade, and histopathologic type have been incorporated in esophageal cancer staging for the first time. However, the roles of tumor location and histologic grade in the survival of esophageal cancer patients are controversial in the existing literature. For example, histologic grade has been reported to be a strong predictive factor of overall survival in several past studies [21, 22]. In contrast, Wijnhoven *et al*. found tumor differentiation to be a prognostic factor according to a univariate analysis but not a Cox regression multivariate analysis [23]. In considering data from other squamous cell carcinoma-predominant databases, Roder *et al*. found that tumor grade was not significantly related to survival [24]. In our study, meanwhile, low grades (i.e., well differentiated tumors) were associated with better overall survival.

As for the cancer location, the results of past studies have also been conflicting [9, 25, 26]. Although some studies have shown that lower third esophageal cancer patients had better prognoses due to increased sufficiency of surgical resection, these studies included a large portion of esophageal adenocarcinoma patients with tumors located in the distal intra-abdominal esophagus; thus, the results may not reflect the precise effects of different tumor locations in ESCC patients [25, 27]. Another study, in which 92% of the cases consisted of squamous cell carcinoma, revealed that 5-year disease-free survival was similar in cases of upper, middle, and lower esophageal cancers [28]. In our study, tumor location was associated with PFS and OS in ESCC with celiac LN metastasis, with lower third ESCC patients having superior PFS and OS compared to those with tumors located in the upper or middle third of the esophagus. These findings suggest that celiac LN metastasis should possibly be viewed as regional LN metastasis in cases of lower third ESCC but as non-regional LN metastasis for ESCC patients with tumors located in the upper or middle third of the esophagus. On the other hand, the lower third ESCC patients in this study had a higher percentage of salvage surgery compared to those with upper or middle third esophageal cancer, resulting in relatively prolonged overall survival. In the 6^th^ edition AJCC staging system, celiac LNs are defined as non-regional LNs in cases of thoracic esophageal cancer, in addition to being classified as M1a stage LNs in cases of lower third esophageal cancer and as M1b stage LNs in cases of upper and middle third esophageal cancer [13]. However, the 7^th^ AJCC staging system re-defines celiac LNs as regional LNs and removes the M1a and M1b classifications [14]. Furthermore, N stages are subclassified based on the absolute number of positive LNs instead of the presence of regional LN involvement. Recently, 8^th^ AJCC staging system has been developed. Although 8th edition of the AJCC staging of esophageal cancer presents separate classifications for clinical, pathologic, and postneoadjuvant stage groups, it also defines celiac LNs as regional LNs, the same as the 7^th^ AJCC staging system [29]. Given these results of our study, the 6^th^ AJCC staging system appears to be more favorable than the 7^th^ or 8^th^ edition in terms of the definition of celiac LN metastasis for locally advanced ESCC patients receiving curative CCRT.

Our study had several limitations. First, it was a retrospective study of patients treated at a single institution, and the sample size was relatively small. Second, there were limited number of patients in the non-celiac LN metastasis group, such that the parameter of sex was not matched well when using the propensity score matching method (that said, sex is not commonly regarded as a prognostic factor of overall survival in the literature). Nonetheless, to the best of our knowledge, this study constitutes the largest series of ESCC patients with celiac LN metastasis who underwent curative CCRT thus far and may thus be useful for understanding this rare disease entity.

In conclusion, the results of our study suggest that celiac LN metastasis is a prognostic factor for locally advanced ESCC patients receiving curative CCRT. Among these ESCC patients with celiac LN metastasis, tumor location is a strongly prognostic factor, indicating that patients with lower third ESCC have better PFS and OS than those with upper or middle third ESCC. For patients with lower third ESCC, there were no differences in treatment outcome between patient with and without celiac LN. For patients with upper or middle third ESCC, the prognosis of patients with celiac LN is worse than that of patients without celiac LN. The 6^th^ AJCC staging system appears to be more favorable than the 7^th^ edition in terms of the definition of celiac LN metastasis for locally advanced ESCC patients receiving curative CCRT.

## MATERIALS AND METHODS

### Patient selection

The records of a total of 1,045 patients with ESCC who were treated at Kaohsiung Chang Gung Memorial Hospital between January 2000 and December 2015 were retrospectively reviewed. Of these 1,045 ESCC patients, we first excluded those patients with a history of second primary malignancy, and then excluded any who did not have stage III disease. After that, only those ESCC patients who received CCRT as a curative treatment were included. Finally, a total of 375 ESCC patients were selected. Of these 375 stage III locally advanced ESCC patients, 51 who had celiac LN metastasis were identified. Therefore, these 375 stage III locally advanced ESCC patients were divided into two groups: one group consisting of the 51 patients with celiac LN metastasis and another consisting of the 324 patients without celiac LN metastasis. Any patients who underwent other therapeutic protocols, such as surgical resection followed by chemotherapy/radiotherapy, palliative chemotherapy, or supportive care, were excluded.

**Figure 5:**
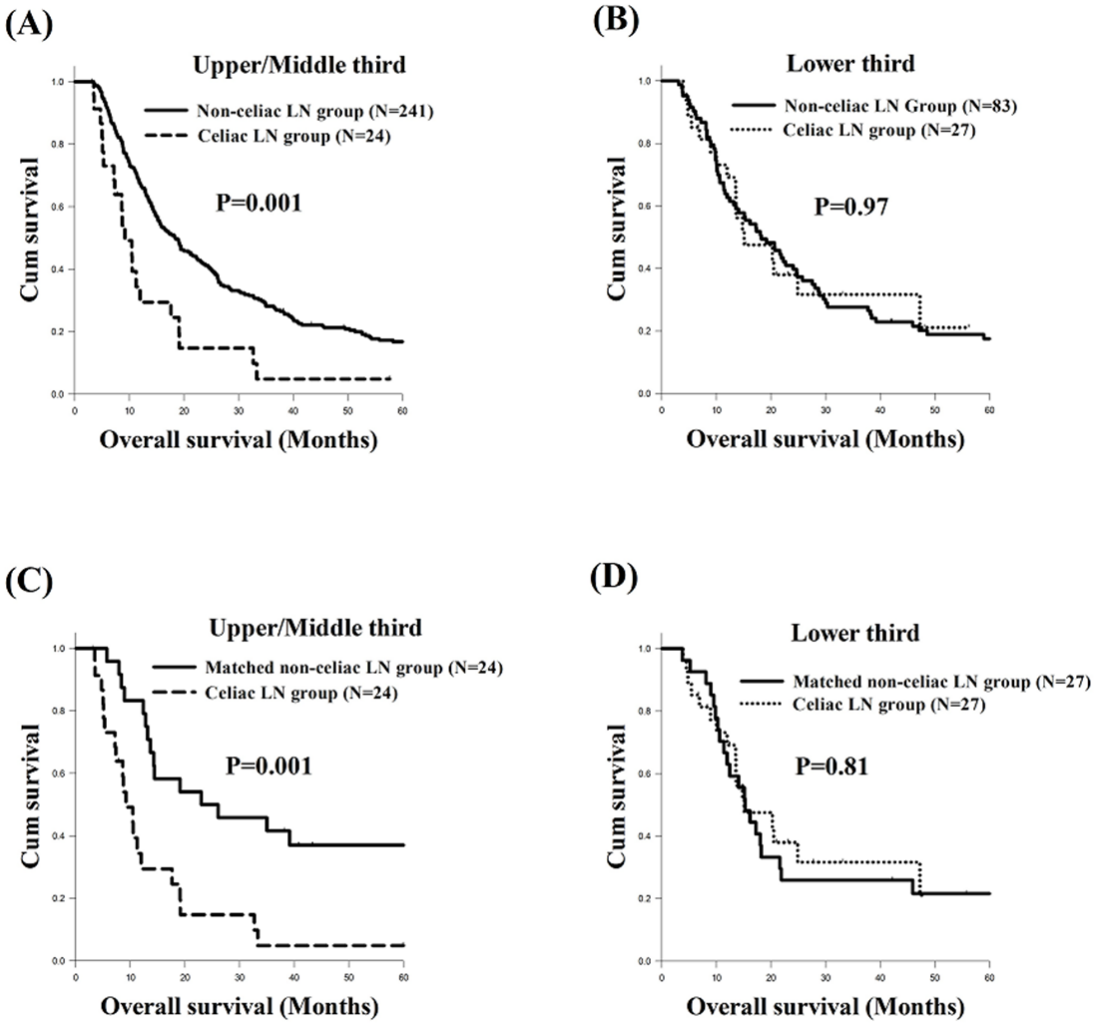
Algorithm for identifying locally advanced stage III ESCC patients with or without celiac lymph node metastasis.

Among the 324 ESCC patients without celiac LN metastasis, the propensity score matching method was used to prevent selection bias. We used binary logistic regression to calculate a propensity score, and the covariates entered in the propensity model were age, sex, tumor stage, tumor grade, and tumor location. Subsequently, a 1-to-1 match between the 51 patients with celiac LN metastasis and 51 patients without celiac LN metastasis was obtained using the closest matching scores, with the 51 matched ESCC patients without celiac LN metastasis being considered as a control group for those with celiac LN metastasis. The algorithm used is shown in Figure 5.

The tumor stages were determined according to the 7^th^ AJCC staging system.

### Determination of clinical tumor stage and identification/definition of celiac lymph nodes

The clinical tumor stage of each case of ESCC was determined by chest CT, EUS, and PET scans. Celiac LNs were defined as those LNs situated around the celiac artery, deeply buried in an almost tunnel-like retroperitoneal location high in the epigastrium, and distinct from left gastric, splenic, or hepatic LNs. LNs were considered metastasis-positive if, first, they were spherical and larger than 10mm in maximum transverse diameter on a chest CT scan or, second, if they were detected to exhibit focal major 18-fluorodeoxy glucose (^18^F-FDG) uptake compared to normal mediastinal activity according to a PET scan.

### Concurrent chemoradiotherapy planning

For local radiotherapy (RT), the patients were simulated using CT-simulators with thermoplastic immobilization devices and were treated using the three-dimensional conformal radiotherapy (3D-CRT) technique or intensity-modulated radiotherapy (IMRT) technique with 6- or 10-MV photons. The gross target volume (GTV) for RT was defined as the gross tumor and gross LNs seen on CT scan and/or PET-CT images. The clinical target volume (CTV) included the whole esophagus, the mediastinal LNs, and the celiac LNs. The planning target volume (PTV) was expanded from the CTV volume with 1.0-2.0 cm margins in all directions. The total dose to the PTV was 50-50.4 Gy in 25-28 daily fractions administered 5 days per week.

Chemotherapy was performed concurrently with radiotherapy, and consisted of cisplatin (75 mg/m^2^; 4-hour drip) on day 1 and 5-fluorouracil (1000mg/m^2^; continuous infusion) on days 1-4 every 4 weeks. Carboplatin was prescribed instead of cisplatin for patients with creatinine clearance < 60 mL/min.

### Statistical analysis

Statistical analyses were performed using the SPSS 19 software package (IBM, Armonk, NY). The chi-square test, Fisher's exact test, and *t*-test were used to compare data between the two groups. PFS was calculated from the date of starting treatment of the esophageal cancer to the date of disease progression or death from any cause, and OS was calculated from the date of diagnosis of the esophageal cancer to the date of death as a result of all causes or to the date of the last follow-up.

The estimated overall survival was calculated using the Kaplan–Meier method, and the differences between groups were assessed using the log rank test for univariate analysis. Multivariate analyses of the prognostic factors for survival were performed using the Cox proportional hazards model. The hazard ratio with 95% confidence interval and P values were calculated to quantify the strength of the associations between the prognostic parameters and survival. For all analyses, two-sided tests of significance were used, with P < 0.05 considered significant.

### Ethics statement

The retrospective analysis was approved by the Chang Gung Medical Foundation Institutional Review Board (201700206B0). All the methods were carried out in accordance with the approved guidelines, and written informed consent of the patients or their families was not judged necessary for this kind of retrospective study by the Chang Gung Medical Foundation Institutional Review Board.

## Abbreviations

LN: lymph node
ESCC: esophageal squamous cell carcinoma
CCRT: curative concurrent chemoradiotherapy
AJCC: American Joint Committee on Cancer
PFS: progression-free survival
OS: overall survival
CT: computed tomography
EUS: endoscopic ultrasonography
PET: positron emission tomography
^18^F-FDG: 18-fluorodeoxy glucose
RT: radiotherapy
IMRT: intensity-modulated radiotherapy
GTV: gross target volume
CTV: clinical target volume
PTV: planning target volume
